# Humanin Rescues Cultured Rat Cortical Neurons from NMDA-Induced Toxicity Not by NMDA Receptor

**DOI:** 10.1155/2014/341529

**Published:** 2014-05-19

**Authors:** Ai-Ling Cui, Jian-Zhong Li, Zhi-Bo Feng, Guo-Lin Ma, Liang Gong, Chun-Ling Li, Ce Zhang, Kefeng Li

**Affiliations:** ^1^Key Laboratory of Tissue Regeneration of Henan Province, Xinxiang Medical College, Eastern Part of Jinsui Road, Xinxiang, Henan 453003, China; ^2^Clinical Laboratory of Heji Hospital Affiliated to Changzhi Medical College, 271 East Taihang Road, Changzhi, Shanxi 046000, China; ^3^Department of Radiology, China-Japan Friendship Hospital, Beijing 100029, China; ^4^Department of Physiology, Shanxi Medical University, No. 56 Xinjian Road, Taiyuan, Shanxi 030001, China; ^5^School of Medicine, University of California, San Diego (UCSD), San Diego, CA 92093, USA

## Abstract

Excitatory neurotoxicity has been implicated in many pathological situations and there is no effective treatment available. Humanin is a 24-aa peptide cloned from the brain of patients with Alzheimer's disease (AD). In the present study, excitatory toxicity was induced by N-methyl-D-aspartate (NMDA) in primarily cultured rat cortical neurons. MTT assessment, lactate dehydrogenase (LDH) release, and calcein staining were employed to evaluate the protective activity of humanin on NMDA induced toxicity. The results suggested that NMDA (100 **μ**mol/L, 2.5 hr) triggered neuronal morphological changes, lactate dehydrogenase (LDH) release (166% of the control), reduction of cell viability (about 50% of the control), and the decrease of living cell density (about 50% of the control). When pretreated with humanin, the toxicity was suppressed. The living cells' density of humanin treated group was similar to that of control. The cell viability was attenuated dose-dependently (IC_50_ = 0.132 nmol/L). The LDH release was also neutralized in a dose-dependent manner. In addition, the intracellular Ca^2+^ overloading triggered by NMDA reverted quickly and humanin could not inhibit it. These findings indicate that humanin can rescue cortical neurons from NMDA-induced toxicity in rat but not through interfering with NMDA receptor directly.

## 1. Introduction

Glutamate has been recognized as a major neurotransmitter and mediates excitatory synaptic transmission in the mammalian central nervous system. Prolonged stimulation with glutamate has been reported to cause neurological disorders [1]. Glutamate plays its roles after binding to the receptors. N-Methyl-D-aspartate (NMDA) receptors, a group of ionotropic receptors of glutamate, are believed to be the key factors to initiate glutamate-induced excitotoxicity [2, 3]. As Ca^2+^  channels, the overactivation of NMDA receptors leads to the overload of Ca^2+^   in the neurons. Although intracellular Ca^2+^   is necessary for nervous physiological processes, Ca^2+^   overload triggers abnormal activation of Ca^2+^   dependent protease, mitochondrial dysfunction, and subsequent neuronal death, which is named excitatory neurotoxicity. Excitatory neurotoxicity has been reported to be involved in a variety of pathogenesis, such as stroke, traumatic brain injury, schizophrenia, Parkinson's disease, cerebral ischemia, and neurodegeneration [4–6]. NMDA receptor antagonists have been explored for many years as therapeutic agents for the treatment of neurological disorders such as stroke, epilepsy, pain, and Parkinson's disease. However, it has been discovered that many of these compounds can cause adverse behavioral effects and produce neurotoxicity [4, 7].

Humanin (HN) is a 24-aa peptide encoded by an identified gene cloned from occipital lobe of the patients with Alzheimer's disease (AD) in autopsy in 2001. It is considered as Alzheimer's disease-selective neuroprotective peptide [8–10]. However, HN does not inhibit other toxic insults to neurons such as the neurotoxicity triggered by Fas, etoposide [8], glutamate [9], and NMDA [11].

Although HN is firstly identified from human brain with Alzheimer's disease, several HN homologues have been discovered in other species of animals including rat, mouse, monkey, and nematode [11–13]. It has also been identified in other tissues besides brain including testis, colon, skeletal muscles, and human vascular walls [13–15]. The widespread of HN implies that its role is versatile other than just attenuating Alzheimer's disease-related insults. In support of this notion, several studies have been conducted to decipher the neuroprotective mechanism of HN at cellular and molecular level, (1) to elevate ATP level [14, 16], (2) to block apoptosis [9, 12, 17, 18], and (3) to interfere with intracellular Ca^2+^   accumulation [19, 20]. The mechanism mentioned above is necessary for any cells to survive under any stresses, which implies that HN might play versatile protective roles against broad insults other than as an AD-selective neuroprotective peptide.

In this study, in order to verify the versatile neuroprotective role of HN, we investigated the effect of HN on NMDA induced neurotoxicity. Our results showed that HN attenuated NMDA-induced neurotoxicity and rescued cultured rat cortical neurons. We also found that HN did not play its neuroprotective role by interfering NMDA receptor directly.

## 2. Materials and Methods

### 2.1. Primary Cerebral Cortical Neuron Culture

The study was based on primarily cultured cortical neurons from neonatal Wistar rat (P1-3). Briefly, the pups were decapitated and cerebral cortexes were isolated and immersed into ice-cold D-Hanks buffer containing (in mM) NaCl 136.7, KCl 5.4, NaHCO_3_ 4.2, KH_2_PO_4_ 0.4, NaH_2_PO_4_ 0.6, glucose 5.6, and pH 7.4. To dissociate cortices into single cells, they were minced mechanically into grains at about 1 mm^3^. The minced cortices were then digested with trypsin (0.03%, pH 7.4, 37°C, Sigma) for 1 min, centrifuged at 2,500 rpm for 5 min and then resuspended in Dulbecco's Modified Eagle's Medium (DMEM, Gibco) with fetal calf serum (FCS, Sigma) 20% (V/V) and penicillin-streptomycin 100 U/mL. Cortical cells were plated on poly-D-lysine coated 6-well (3 mL) or 96-well (100 *μ*L) plates (Costar) at 5 × 10^5^ cells/mL. Cells on 6-well plates with coverslips were used for calcein staining and Ca^2+^ instantaneous concentration. Cells without coverslips were used for the assay of lactate dehydrogenase (LDH) release. Cells grown on 96-well plates were used for MTT assay. Cytosine arabinoside (Ara-C) (Sigma) (10 *μ*mol/L) was added 24 hr after cell plating to limit the proliferation of nonneuronal cells. The culture media were half-refreshed every 2 days. All experiments were performed at DIV9.

### 2.2. Experimental Groups

In this study, the cultured neurons were grouped as follows: (1) control group, cultured neurons only; (2) HN group, cultured neurons incubated with HN (10 *μ*mol/L); (3) NMDA group, cultured neurons incubated with NMDA (100 *μ*mol/L) and Glycine (10 *μ*mol/L) for 2.5 hr; (4) MK-801 group, MK-801 (10 *μ*mol/L) was added into NMDA group; (5) NMDA^+^ + HN group, HN was added into NMDA treated cells to achieve the final concentration of 0.01 *μ*mol/L, 0.1 *μ*mol/L, 1 *μ*mol/L, 10 *μ*mol/L, and 100 *μ*mol/L.

### 2.3. Chemicals Treatment

Cortical neurons isolated from the pups were cultured for 8 days before chemical treatment. HN (Shanghai Shenggong, China) was administrated 16 h in advance before NMDA treatment. After being preincubated with or without HN as grouped, neurotoxicity was induced by the treatment of 100 *μ*mol/L of NMDA as described [21]. Acute neurotoxic exposure (NMDA challenge) was achieved by removing culture medium from cultured neurons. Collected medium was filter-sterilized and stored for future analysis. To remove traces of growth medium, cells were washed three times with prewarmed Locke's buffer containing (in mM) NaCl 154, KCl 5.6, NaHCO_3_ 3.6, CaCl_2_ 2.3, MgCl_2_ 1.2, glucose 5.6, HEPES 5, and pH 7.4. They were incubated for 2.5 hr with either drug-free or neurotoxin-containing Mg^2+^ free Locke's buffer (NMDA and MK-801/HN were added at concentrations as mentioned above). The incubation was terminated by removal of drug-containing buffer, followed by washing with prewarmed drug-free Locke's buffer containing 1 mM Mg^2+^. Finally, cells were cultured in previously collected original culture medium. At 24 hr, cells on coverslips were subjected to the assay of calcein staining and intracellular Ca^2+^   concentration measurement. Cells on 96-well plates were used for MTT assay. The medium in 6-well plates without coverslips was collected for the measurement of LDH release.

### 2.4. MTT Assay

Methylthiazolyldiphenyl-tetrazolium bromide (MTT) assay is a method to evaluate cell viability by measuring the integrity of mitochondria of viable cells. The assay was performed 24 hr after the NMDA treatment as described [22]. It was initiated by removing the old culture medium and adding MTT dissolved in serum-free culture medium (final concentration of 0.5 mg/mL, Sigma). Following 4 hr incubation at 37°C, the medium was aspirated and 0.1 mL of dimethyl sulfoxide was added to lyse the cells and dissolve the formazan crystals. The absorbance was recorded at 490 nm in a microplate reader (Benchmark, Bio-Rad). Cell viability was expressed as percentage of the absorption in control group which is exposed to drug-free buffer (100%).

### 2.5. Calcein Staining

Calcein-acetoxymethyl ester (calcein-AM) is a nonfluorescent highly lipophilic and cell membrane permeant compound, which can be converted by intracellular esterases into calcein, an anionic fluorescent form. Therefore, calcein-AM only stains viable cells and provides both morphological and functional information of viable cells under microscopy and fluorometry. In this experiment, calcein staining was performed as described [23]. In brief, at 24 h after NMDA exposure, cells were washed once in Locke's buffer and loaded with 5 *μ*M calcein-AM (Fluka) in Locke's buffer for 30 min. The neurons were then fixed in 4% paraformaldehyde for 30 min and fluorescence was visualized under fluorescent microscope (Olympus BX51) using 488 nm excitation and 520 nm emission filters. Five to eight independent experiments were performed and 4-5 random 200x fields per coverslip were photographed in each observation. Cell numbers were counted in each field. The data was shown as the percentage of fluorescent cells in each group compared with control.

### 2.6. Determination of LDH Release

Cell damage was quantified by the measurement of LDH activity in the culture medium released from the damaged cells. In the experiment, the culture mediums of the 6-well plates were collected at 24 h after NMDA exposure. The activity of LDH in the culture medium was measured as described by Koh and Choi [24]. The release values were expressed as the percentage compared with that of control group which was set as 100%. The data presented were mean ± SD for 5 to 8 independent experiments using different batches of cells.

### 2.7. Measurement of Intracellular Ca^2+^ Concentration

At DIV 8, the cultured neurons on coverslips were given HN according to the experimental group. After 16 h, all groups were washed 3 times in Locke's buffer. They were then loaded with Fluo 3/AM (Biotium, Hayward, USA) in Locke's buffer containing 5 *μ*mol/L Fluo 3/AM away from light and incubated at 37°C for 40 min. The neurons were then washed 3 times with D-Hanks and observed under confocal laser scanning microscope (CLSM, Leica TCS SP5 II). Strong single neuron in the field was enrolled to be the subject. Ca^2+^   dependent fluorescence intensity was measured at an excitation wavelength of 488 nm and an emission wavelength of 530 nm. The parameter was set to PMT 850V and 30% of the intensity. Dynamic changes of cytoplasmic Ca^2+^   were captured and recorded every 660 ms and total 300 successive measurements (for about 198 s) were recorded for each subject. Five to ten neurons were measured in each group and the average intensity was regarded as the intracellular Ca^2+^   instantaneous concentration of each group.

### 2.8. Statistical Analysis

All data were expressed as the mean ± SEM. Significance of difference between groups was tested using one-way analysis of variance (ANOVA) followed by post hoc Tukey's multiple comparison test. *P*values less than 0.05 were considered to be significant.

## 3. Results

### 3.1. NMDA Induced Occurrence of the Morphological Changes of Cortical Neurons

**Figure 1:**
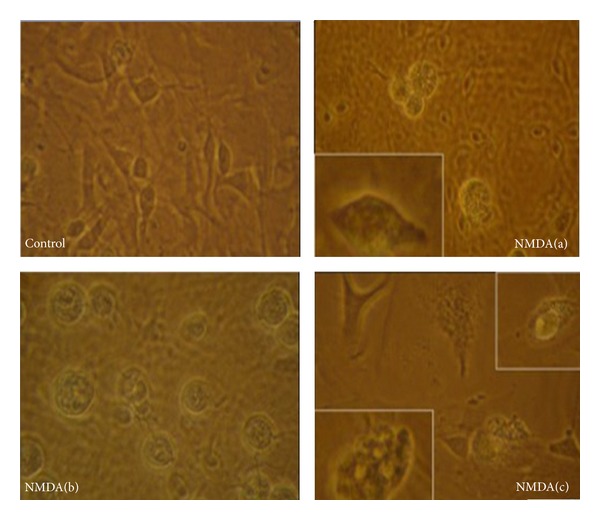
Morphological changes of cortical neurons induced by NMDA. Representative images of control and NMDA-treated cortical neurons (a, b, and c) were observed under phase-contrast light microscope.

To establish a model of excitatory neurotoxicity, we treated cultured neurons at DIV9 with NMDA and observed the morphological changes under phase-contrast light microscope. Neurons at control group adhered to the plate bottom with clear nucleus and stretched with many processes. NMDA (100 *μ*mol/L) treatment for 2.5 hr induced the retraction of processes (Figure 1(b)). Subsequently, neurons turn round and their classical morphological characteristics disappeared (Figure 1(a)). Later, thick particles could be discerned in the damaged cells (Figure 1(c)) and then the bodies were burst into pieces. The remnant could still be seen in the picture (Figure 1(c)). The changes of cell morphology indicated the successful induction of excitatory neurotoxicity by 100 *μ*mol/L of NMDA.

### 3.2. Humanin Blocked the Reduction of Living Neurons Evoked by NMDA

**Figure 2:**
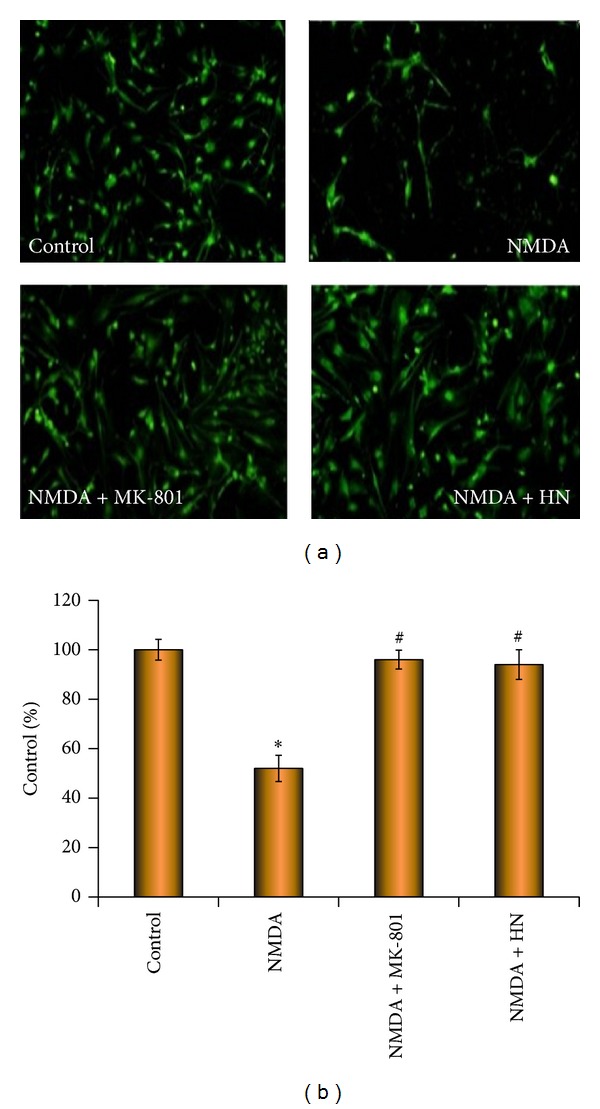
Effect of humanin on density of living neurons under the challenge of NMDA. Representative cellular density of each group is shown by calcein staining under fluorescence microscope. Each bar represents mean ± SEM of eight independent observations. The date from each observation is expressed as the percentage of fluorescent cells in each group compared with control (100%). Statistical significance is at *P* < 0.05. *F*_(3,116)_ = 4.826 and *P* = 0.003 according to one-way ANOVA analysis. ∗ represents control versus NMDA group, *P* = 0.012; # represents NMDA group versus NMDA + MK-801 group (*P* = 0.026) or NMDA + HN (Humanin) group (*P* = 0.009). ∗ and # mean the existence of statistical significance between.

Calcein staining was employed to evaluate the density of living neurons. NMDA (100 *μ*mol/L, 2.5 hr) induced a significant decrease of living neurons in NMDA group. About half of the cells died. MK-801 is a noncompetitive antagonist of NMDA receptor. In MK-801 group, the density of living neurons was similar to the control which indicated that the toxicity was induced by NMDA. There was no statistical difference on the density of living neurons between HN group and control group, suggesting that HN (100 *μ*mol/L) could mimic the function of MK-801 in blocking neuron toxicity triggered by NMDA (see Figure 2).

### 3.3. Humanin Restored Cell Viability Decreased by NMDA Treatment

**Figure 3:**
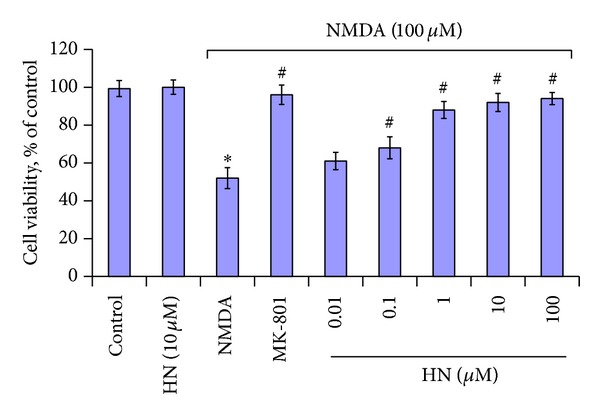
Effect of humanin on NMDA triggered reduction of cell viability in cortical neurons as measured by MTT cell viability assay. Each bar represents mean ± SEM of five independent observations. Statistical significance is at *P* < 0.05. *F*_(8,64)_ = 2.697 and *P* = 0.013 according to one-way ANOVA analysis. ∗ represents control versus NMDA group (*P* = 0.032); control versus HN (10 *μ*mol/L), *P* = 0.421; # represents NMDA group versus NMDA + MK-801 group (*P* = 0.026) or NMDA + HN (Humanin) group. HN (0.01 *μ*mol/L), *P* = 0.289; HN (0.1 *μ*mol/L), *P* = 0.036; HN (1 *μ*mol/L), *P* = 0.024; HN (10 *μ*mol/L), *P* = 0.012; HN (100 *μ*mol/L), *P* = 0.021. ∗ and # mean that there is statistical significance between control and treatment groups.

NMDA (100 *μ*mol/L, 2.5 hr) was toxic to cortical neurons and caused the decrease of cell viability to 50% of the control as measured by MTT (Figure 3). The cell viability of MK-801 group was similar to that in control, which confirmed that the toxicity was evoked by NMDA. HN itself in the medium did not inhibit the cell viability. The cell viability was recovered by the addition of HN in NMDA treatment group. When the concentration of HN reached 0.1 *μ*mol/L, the decrease of cell viability began to be attenuated in NMDA treatment group. Ninety-eight percentage of the cell viability was recovered when 10 *μ*mol/L of HN was added. Our results indicated that HN attenuated NMDA-induced insults effectively and dose-dependently (IC_50_   was 0.132 *μ*mol/L).

### 3.4. Humanin Reduced the Release of LDH Triggered by NMDA

**Figure 4:**
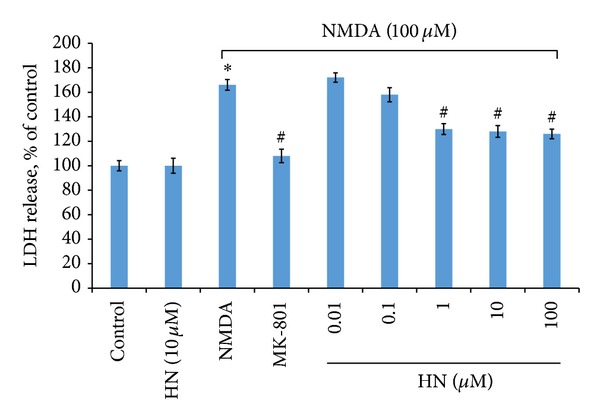
Effect of humanin on NMDA triggered LDH release in cultured cortical neurons. Each bar represents mean ± SEM of 5–8 independent observations. Statistical significance is at *P* < 0.05. *F*_(8,64)_ = 3.023 and *P* = 0.006 after one-way ANOVA analysis in SPSS. ∗ represents control versus NMDA group (*P* = 0.028); # represents NMDA group versus NMDA + MK-801 group (*P* = 0.019) or NMDA + HN (Humanin) group. HN (0.01 *μ*mol/L), *P* = 0.384; HN (0.1 *μ*mol/L), *P* = 0.301; HN (1 *μ*mol/L), *P* = 0.039; HN (10 *μ*mol/L), *P* = 0.036; HN (100 *μ*mol/L), *P* = 0.040. ∗ and # mean that there is statistical significance between control and treatment groups.

Our results showed that NMDA treatment (100 *μ*mol/L, 2.5 hr) caused the damage of cultured neurons and thus triggered the overrelease of LDH (Figure 4). LDH concentration in NMDA treatment group was about 1.66-fold higher than that in the control. MK-801 is a noncompetitive antagonist of NMDA receptor. As shown in Figure 4, the addition of MK-801 significantly reduced the release of LDH in NMDA treated neurons. The effect of HN is similar to MK-801. Our results demonstrated that HN itself had no effect on normal neurons. However, the release of LDH was significantly inhibited in NMDA treated cells when the neurons were pretreated with HN for 16 h. The concentration of LDH was about 135% of the control when 1 *μ*mol/L of HN was added. No additional effect, however, could be detected when the concentration of HN continuously increased, even reached 100 *μ*mol/L.

### 3.5. HN Inhibited the Increase of Cytoplasmic Ca^2+^ Concentration Induced by NMDA

**Figure 5:**
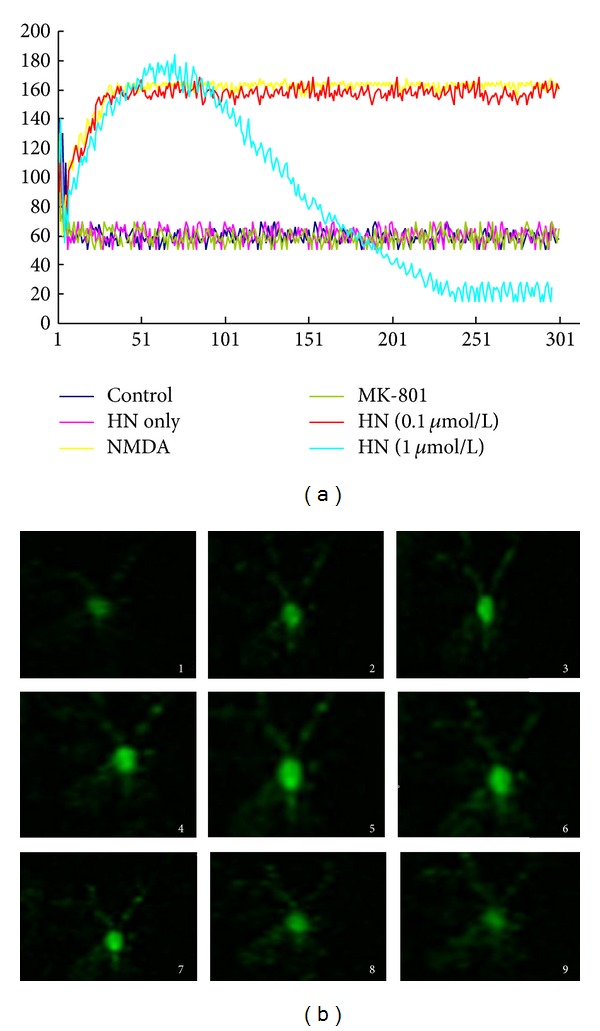
(a) Effect of humanin on NMDA triggered dynamic changes of cytoplasmic Ca^2+^ concentration. The fluorescence intensity which is related to cytoplasmic Ca^2+^ concentration was captured and recorded every 660 ms for 300 successive measurements (for about 198 s) for each subject. 5 to 10 neurons were measured in every group. The curves are formed by the connection of 300 data which stand for the average instantaneous fluorescence intensity of each group. HN only: the addition of 10 *μ*M HN itself in the medium. HN (0.1 *μ*mol/L) and HN (1 *μ*mol/L) groups: HN (0.1 *μ*mol/L) or HN (1 *μ*mol/L) in NMDA treatment groups. (b) Effect of humanin on NMDA triggered dynamic changes of cytoplasmic Ca^2+^ concentration. Picture 1 to picture 9 stand for the typical successive cellular morphological characteristics of HN (1 *μ*mol/L) on NMDA-triggered increase of cytoplasmic Ca^2+^   concentration.

Our results showed that NMDA (100 *μ*mol/L) triggered the rapid increase of cytoplasmic Ca^2+^ concentration and stayed at the highest level during the observation (Figure 5(a)). It only took 2 s for the increase of cytoplasmic Ca^2+^   concentration from the base level (60) to the peak value (160). HN (0.1 *μ*mol/L) had no effect on interfering with NMDA-induced increase of cytoplasmic Ca^2+^   concentration. Although HN (1 *μ*mol/L) did not block the increase of cytoplasmic Ca^2+^   concentration induced by NMDA, it caused the gradual decrease of Ca^2+^ concentration from the peak value (180) to the control level (60) and even lower than the control (20) within 110 s.

## 4. Discussion

Excitotoxicity is a leading cause of neurodegeneration observed in progressive and acute brain diseases. Despite many years search for effective neuroprotectants, there is still no effective therapy [25]. Humanin was cloned and identified from an apparently normal region of brain with Alzheimer's disease in 2001 and was considered as Alzheimer's disease-selective neuroprotective peptide. But its wide location and the neuroprotective mechanism underlying suggests it to be versatile other than Alzheimer's disease-selective. Moreover, HN is encoded, transcribed, synthesized, and secreted from the occipital lobe of human brain. Therefore, HN might be a promising candidate in the therapy of excitatory neurotoxicity. Based on the consideration mentioned above, the possible neuroprotective role of HN against excitatory neurotoxicity was reverified in our experiment. In this study, the cytotoxicity of NMDA was quantified by measuring the activity of LDH released from the cultured neurons into the medium, and neuronal survival was quantified spectrophotometrically using MTT. The density of living cells was evaluated by calcein staining. Our results showed that NMDA caused the significant decrease of cell viability of cortical neurons. The addition of HN could, however, attenuate NMDA induced toxicity and increase the cell viability efficiently and dose-dependently. The IC_50_ was 132 nmol/L, a much lower concentration compared with that to neutralize Alzheimer's disease-related toxicity [9].

The result was confirmed by calcein staining. NMDA-induced toxicity triggered much more neuronal death in NMDA group. However, HN addition protected the neurons from NMDA-induced cell death. Additionally, NMDA evoked neuronal death and subsequent overrelease of LDH into medium. HN could neutralize the toxicity and reduced the release of LDH although it could not completely block the release.

The cytoprotective function of HN on excitatory neurotoxicity has ever been investigated by two laboratories by far. However, no protective effect was detected in either glutamate-induced [9] or NMDA-triggered [11] neuronal cell loss, which is different from what we have discovered in this study. The difference can be interpreted at least in part by the different experimental models. (1) The toxicity induced was different. In our experiment, the neurotoxicity was caused by NMDA while in Hashimoto's was by glutamate. Although the toxic agent in Caricasole's experiment and ours were the same even at the same dose (NMDA 100 *μ*mol/L), but the period cortical neurons exposed to the toxic agent was different (ours for 2.5 hr versus Caricasole's for 10 min). As toxicant, the longer the neurons were treated, the severer the damage it might have to the neurons; (2) HN was added to the medium 16 h before the exposure of NMDA in our experiment. In Caricasole's study, HN was added 20 h after NMDA treatment (after exposure). The molecular mechanisms of the interaction between humanin and the neuron cells have not been completely understood. It is possible that the pretreatment of humanin activated the antioxidative defense system of the cells and thus protected the cells from NMDA stress [26]. (3) Different assay methods were employed. The toxicity was evaluated by MTT, LDH release, and calcein staining in our experiment, while trypan blue staining was employed to examine the neuronal death in the other two laboratories. Trypan blue can accumulate slowly in living cells and cause the cytotoxicity. The cell viability rate evaluated by trypan blue is usually lower than the real viable cell number.

In our experiment, the results suggested that HN was effective in attenuating NMDA-induced toxicity. But there was difference in effective concentration in the three methods. Because MTT assay is based on succinate dehydrogenase located in mitochondria, it could be positively detected even if the cell membrane was slightly broken. When the neuron cell membrane was completely broken, both MTT and LDH release could be positively detected. Humanin (10 *μ*mol/L) could simulate the action of MK-801 and most recovered cell viability in MTT measurement. However, in the detection of LDH release, humanin could not inhibit the toxicity completely, even the concentration reached 100 *μ*mol/L. In calcein staining, the activity of intracellular esterases was measured. The results were consistent with MTT assay. Each method has its own advantages and disadvantages. It is necessary to evaluate the effect of HN based on the results of several different methods to avoid the limitations of each assay.

Since the toxicity was induced by NMDA in the experiment, overactivation of NMDA receptor and subsequent Ca^2+^   overloading is the toxic resource. How did Humanin interfere with Ca^2+^   overloading? To answer this quest, we measured intracellular Ca^2+^   instantaneous concentration and found that HN could not inhibit intracellular Ca^2+^   overloading triggered by NMDA. However, it could induce the reverting quickly, suggesting that HN attenuated NMDA-induced toxicity not by interfering with NMDA receptor directly. Further study on the expression of calcium handing proteins is needed to confirm the role of HN in calcium dynamics.

It was reported that HN mediates its protective effect by three putative receptors. Cytosolic HN partly inhibits staurosporine-induced cell death by binding to Bax and neutralizing the Bax-mediated proapoptotic pathway [12]. Secreted HN binds to cell-surface formyl peptide receptor-like-1 and a cell-surface receptor that belongs to the interleukin- (IL-) 6 receptor family to inhibit A**β**-induced death of PC12 neuronal cells [27]. It is likely that the addition of HN into the medium will perform the similar role as the secreted HN. In MTT assessment, IC_50_ of HN against NMDA-induced toxicity is about 0.1 *μ*mol/L that is much lower than that of Humanin's protection in the death systems (1–10 *μ*mol/L) mentioned above. This difference suggested that HN's receptor mediating the HN-induced protection against NMDA toxicity to rat cortical neurons is different from the receptor that has been reported in the earlier studies. Attention should be paid to unveil the mechanism underlying before HN could be applied to clinic.

It was reported that HN increased the metabolic rate and the level of ATP in the cells [14, 15]. HN is also expressed in human vascular walls and had a cytoprotective effect against oxidized LDL-induced oxidative stress [26]. Since MTT assessment is based on the activity of mitochondrial dehydrogenases, HN might protect the neurons from NMDA-induced toxicity by promoting the efficiency of mitochondria. A healthier mitochondrion could afford much more power to recover the elevated cytosolic Ca^2+^ concentration. Mitochondria dysfunction is the common characteristic when living cell is under insults. Promoting the efficiency of mitochondria may at least in part interpret the wide location of HN if it is the truth.

## 5. Conclusion

In this study, we employed NMDA induced toxicity as a model of excitatory neurotoxicity to explore the neuroprotective potential of humanin. We found that humanin rescued cortical neurons from NMDA-evoked toxicity effectively in rat in a dose-dependent manner. Our results indicated that humanin was not a specific neuroprotective peptide against Alzheimer's disease. It has broad neuroprotective activities. Our research thus sheds light on the therapy of excitatory neurotoxicity by supporting humanin as a promising candidate. Attention should be paid to unveil the mechanism underlying before humanin could be applied to clinic.
